# High incidence of antimicrobial resistant organisms including extended spectrum beta-lactamase producing Enterobacteriaceae and methicillin-resistant *Staphylococcus aureus *in nasopharyngeal and blood isolates of HIV-infected children from Cape Town, South Africa

**DOI:** 10.1186/1471-2334-8-40

**Published:** 2008-04-01

**Authors:** Mark F Cotton, Elizabeth Wasserman, Juanita Smit, Andrew Whitelaw, Heather J Zar

**Affiliations:** 1 Departments of Paediatrics and Child Health, Faculty of Health Sciences, Stellenbosch University, Cape Town South Africa; 2 Department of Pathology and National Health Laboratory Systems, Faculty of Health Sciences, Stellenbosch University, Cape Town, South Africa; 3 Division of Medical Microbiology, National Health Laboratory Systems, University of Cape Town, Cape Town, South Africa; 4 School of Child and Adolescent Health, University of Cape Town, Cape Town, South Africa

## Abstract

**Background:**

There is little information on nasopharyngeal (NP) flora or bacteremia in HIV-infected children. Our aim was to describe the organisms and antimicrobial resistance patterns in children enrolled in a prospective study comparing daily and three times weekly trimethoprim-sulfamethoxazole (TMP-SMX) and isoniazid (INH) or placebo prophylaxis.

**Methods:**

NP swabs were taken at baseline from HIV-infected children enrolled in the study. Standard microbiological techniques were used. Children were grouped according to previous or current exposure to TMP-SMX and whether enrolled to the study during a period of hospitalization. Blood culture results were also recorded within 12 months of baseline.

**Results:**

Two hundred and three children, median age 1.8 (Interquartile [IQ]: 0.7–4) years had NP swabs submitted for culture. One hundred and eighty-four (90.7%) had either stage B or C HIV disease. One hundred and forty-one (69.8%) were receiving TMP-SMX and 19 (9.4%) were on antiretroviral therapy. The majority, 168 (82%) had a history of hospitalization and 91 (44.8%) were enrolled during a period of hospitalization. Thirty-two subjects (16.2%) died within 12 months of study entry.

One hundred and eighty-one potential pathogens were found in 167 children. The most commonly isolated organisms were *Streptococcus pneumoniae *(48: 22.2%), Gram-negative respiratory organisms (*Haemophilus influenzae *and *Moraxella catarrhalis*) (47: 21.8%), *Staphylococcus aureus *(44: 20.4%), Enterobacteriaceae 32 (14.8%) and Pseudomonas 5 (2.3%).

Resistance to TMP-SMX occurred in > 80% of pathogens except for *M. catarrhalis *(2: 18.2% of tested organisms). TMP-SMX resistance tended to be higher in those receiving it at baseline (p = 0.065). Carriage of Methicillin resistant *S. aureus *(MRSA) was significantly associated with being on TMP-SMX at baseline (p = 0.002). Minimal inhibitory concentrations (MIC) to penicillin were determined for 18 *S. pneumoniae *isolates: 7 (38.9%) were fully sensitive (MIC ≤ 0.06 μg/ml), 9 (50%) had intermediate resistance (MIC 0.12 – 1 μg/ml) and 2 (11.1%) had high level resistance (MIC ≥2 μg/ml). Fifty percent of Enterobacteriaceae produced extended spectrum beta-lactamases (ESBL) (resistant to third generation cephalosporins) and 56% were resistant to gentamicin. Seventy-seven percent of *S. aureus *were MRSA. Carriage of resistant organisms was not associated with hospitalization.

On multivariate logistic regression, risk factors for colonization with Enterobacteriaceae were age ≤ one year (Odds ratio 4.4; 95% Confidence Interval 1.9–10.9; p = 0.0008) and CDC stage C disease (Odds ratio 3.6; 95% Confidence Interval 1.5–8.6; p = 0.005)

Nineteen (9.4%) subjects had 23 episodes of bacteremia. Enterobacteriaceae were most commonly isolated (13 of 25 isolates), of which 6 (46%) produced ESBL and were resistant to gentamicin.

**Conclusion:**

HIV-infected children are colonized with potential pathogens, most of which are resistant to commonly used antibiotics. TMP-SMX resistance is extremely common. Antibiotic resistance is widespread in colonizing organisms and those causing invasive disease. Antibiotic recommendations should take cognizance of resistance patterns. Antibiotics appropriate for ESBL-producing Enterobacteriaceae and MRSA should be used for severely ill HIV-infected children in our region. Further study of antibiotic resistance patterns in HIV-infected children from other areas is needed.

## Background

There are few data on nasopharyngeal (NP) flora from HIV-infected children. Most studies have focused on specific organisms such as *Streptococcus pneumoniae*, *Staphylococcus aureus *and *Haemophilus influenzae*, with provision of limited antibiotic resistance data, mainly to penicillin [1-5]. Although antibiotic resistance has been documented in some of these studies, there is need for more information. Thus far, there are no studies documenting the presence of other potential pathogens.

Colonization of the NP by potential respiratory pathogens *S. pneumoniae*, *H. influenzae, S. aureus *and *Moraxella catarrhalis *is established early in childhood. Factors involving colonization and elimination are not well understood but probably involve adhesive and immunologic factors [6]. NP colonization with Enterobacteriaceae occurs in malnourished children and also those from impoverished environments [7,8]. That NP colonization precedes invasive disease has been well established in the rat model [9], meningococcal meningitis [10] and for *S. pneumoniae *in children [11,2]. Pharyngeal colonization by *Salmonella *species in resource-poor settings has also been linked to invasive disease [12].

There are few data in HIV-infected children on organisms causing bacteremia. Madhi et al documented an increased risk of MRSA and trimethoprim-sulfamethoxazole (TMP-SMX) resistance in HIV-infected compared to uninfected infants with bacteremic community-acquired pneumonia in 2000 [13].

TMP-SMX prophylaxis, given to all HIV-exposed and infected children from 6 weeks of age to prevent *Pneumocystis jirovecii *pneumonia (PCP) has been associated with NP carriage of multiresistant *S. pneumoniae *[14] and increased colonization with *S. aureus *[15]. Moreover, its use for intercurrent infections such as otitis media, is linked to increased resistance [16].

The aim of this study was to describe the baseline bacterial flora and antimicrobial resistance patterns of potentially pathogenic bacteria in HIV-infected children enrolled in a prospective study investigating the long-term effects of TMP-SMX and INH prophylaxis [17]. Secondary aims were to examine the effects of prior TMP-SMX on NP organisms and TMP-SMX resistance and to explore relationships between colonizing flora, nutritional status, age, extent of HIV disease, and hospitalization status. Lastly, blood culture isolates, which represent the most extreme form of invasive disease, were reviewed.

## Methods

### Study population

The study population comprised HIV-infected children aged 8 weeks or older, attending either Red Cross (RCCH) or Tygerberg Children's Hospitals (TCH) in Cape Town and recruited for a study of daily versus thrice weekly TMP-SMX given with Isoniazid (INH) or placebo [17]. The study commenced in December 2002. Due to resource constraints, no HIV-uninfected infants were recruited as controls. The Ethics Committees of Cape Town and Stellenbosch Universities approved the study. Written, informed consent was obtained from a parent or legal guardians prior to enrolment.

At enrolment, note was taken of previous and current hospitalization, concomitant medication, including TMP-SMX and antiretroviral therapy. According to baseline TMP-SMX prophylaxis status, subjects were grouped as follows: 1) no prophylaxis, 2) currently receiving TMP-SMX 3) TMP-SMX not currently used but given in the past. Race was not documented in subjects. All came from socio-economically deprived settings and attended public healthcare facilities. The median household income was $126 per month, with on average 5 persons per household, translating into $0.84/person/day and below the monthly minimum subsistence of $133 defined by the South African Government. Nearly half (44%) of children lived in informal settlements [18].

Subjects were classified for severity of HIV disease and CD4 cell depletion according to the Centres for Disease Control and Prevention (CDC) classification system. [19] Weight for age Z-score was calculated using Epi-Info 2004, CDC, Atlanta, GA. Mortality data for the first 52 weeks on the study was extracted from the trial database. Blood culture data were obtained from the National Health Laboratory databases at the two hospitals from a week prior until 52 weeks post enrolment.

### Laboratory methods

NP swabs, (urethral/ENT straight wire swabs transported in Amies charcoal media (Medical wire & equipment, Wiltshire, U.K) were collected at baseline according to a prescribed protocol. A single microbiologist processed all specimens. Swabs were inoculated on appropriate media to isolate staphylococci, streptococci (including *S. pneumoniae*), *H. influenzae, M. catarrhalis *and other Gram-negative organisms. After incubation, organisms were identified according to routine laboratory procedures. Susceptibility testing (Kirby-Bauer disc diffusion) was performed on all isolates and interpreted according to Clinical Laboratory Standards Institute (CLSI) [20]. For *M. catarrhalis*, β-lactamase production was determined using a nitrocephin method (Oxoid, Hampshire, England). The prescribed quality control strains of the American Type Culture Collection (ATCC) were used to verify all susceptibility tests. Extended spectrum beta-lactamase (ESBL) production was detected using the double disc diffusion test, by placing a disc with amoxicillin/clavulanate adjacent to discs with cefotaxime and ceftazidime (1 disc with each), and looking for synergy between the clavulanic acid and the cephalosporin [21]. MRSA was detected using oxacillin discs on salt agar [22].

Isolates of *S. pneumoniae*, *H. influenzae *and *M. catarrhalis *were stored at -70°C in a glycerol nutrient broth enriched with 5% horse blood. Broth dilution MIC's were performed in batches according to CLSI methodology. A single microbiologist (JS) processed all NP swabs.

*M. catarrhalis *and *H. influenzae *were categorized as Gram-negative respiratory organisms and all Enterobacteriaceae were grouped together. Subjects with no growth, contaminated cultures, coagulase negative staphylococci or only commensals were also combined into a single group.

#### Statistics

Descriptive statistics used to summarize ordinal and continuous variables were the median and interquartile range (IQR). The chi-square and Fisher's exact tests were used for analysis of categorical data and the Kruskal-Wallis test was used for non-parametric one-way analysis of variance. A multiple logistic regression model was used to determine risk factors for colonization by predominant organisms. Odds ratios and 95% confidence intervals were reported. The model included all factors from bivariate analyses evaluated a significance level of 0.1. Analyses with performed using JMP 5.1 (SAS, Cary, CA, USA).

## Results

NP cultures were submitted for 203 children. Baseline demographics are shown in Table 1. Median age was 1.8 (IQ 0.7 – 4 years. The majority (89.7%) of children had moderate or severe HIV disease and 78% had CD4 cell depletion. The median weight for age-Z score (WAZ) was -1.6. Almost 70% were receiving TMP-SMX at baseline. The majority of subjects had been hospitalized previously, with only 38 subjects (18.7%) never having been hospitalized. Almost half (44.8%) were recruited during a period of hospitalization. Nineteen (9.4%) children were on antiretroviral therapy (ART). Thirty-five children (17.2%) died within 12 months of enrolment.

**Table 1:** Baseline demographic data in HIV-infected children with NP specimens

	**Patients **N = 203
Female	96 (47.3%)

Median age (IQ range)	1.8 (0.7 – 4) years

CDC Clinical Class	
N	2 (0.9%)
A	17 (8.4%)
B	138 (68%)
C	46 (22.7%)

CDC Immunological	
1	44 (22%)
2	85 (42.5%)
3	71 (35.5%)

CD4 percentage	19 (14 – 26)

Hospitalization	
None in past	52 (25.6%)
1	54 (26.6%)
2	53 (26.11%)
3	28 (13.79%)
4	11 (5.42%)
5	5 (2.46%)
Recruited to study during or immediately after current hospitalization	91 (44.8%)
No hospitalization	38 (18.7%)

TMP-SMX status	

No previous experience	47 (23.3%)
On drug at baseline	141 (69.8)
History of exposure	15 (7.4%)

Median weight for age Z-score (IQ range)	-1.6 (-3 – -0.4)
Antiretroviral therapy at baseline	19 (9.4%)
Time on therapy	0.5 (0.2 – 1.1) years

Died within 12 months of enrolment	35 (17.2%)

Infants without prior TMP-SMX were significantly younger than those on TMP-SMX at baseline (1.3 [IQ: 0.2–2.1] versus1.9 [IQ: 0.4–4.4] years) or than those previously on TMP-SMX (3.2 [IQ: 1.8–6.3 years) (p = 0.0008). NP organisms and TMP-SMX status are shown in Table 2. One hundred and eighty-one potential pathogens were isolated from 167 subjects. Five had two organisms within a group, such as *H. influenzae *and *M. catarrhalis *or different species of Gram-negative Enterobacteriaceae. The most common organisms were *S. pneumoniae *(48: 22%), Gram-negative respiratory organisms (*H. influenzae *and *M. catarrhalis*) (47: 21.7%), *S. aureus *(44: 20.4%), and Enterobacteriaceae (32: 14.1%). There were no significant differences in organisms isolated by TMP-SMX status, except for *P. aeruginosa *and *Acinetobacter *which were more common in subjects not on TMP-SMX at baseline (p = 0.01 and 0.048, respectively).

**Table 2:** Pathogenic NP organisms and TMP-SMX status at baseline

	**Total**	**Not on TMP-SMX at baseline**	**On TMP-SMX at baseline**	**Previously received TMP-SMX**
**†Pathogenic organisms**				
*S. pneumoniae*	48 (26.5%)	9 (5%)	36 (19.9%)	3 (1.7%)
§*Gram negative respiratory organism *(*M. catarrhalis, H. influenzae*)	48 (26.5%)	7 (3.2%)	35 (19.3%)	6 (3.3)
*S. aureus*	44 (24.3%)	10 (5.5%)	31 (17.1%)	3 (1.7%)
Enterobacteriacae	32 (17.7%)	13 (9.9%)	18 (9.9%)	1 (0.5%)
Non-fermenters including *Pseudomonas aeruginosa #*	5 (2.8%)	4 (2.2%)	1 (0.5%)	0
*Acinetobacter *species ¶	4 (2.2%)	3 (1.7%)	1 (0.5%)	0

**† **10 subjects had 2 organisms and 2 had 3 organisms isolated from a NP swab.

§*M. catarrhalis *n – 22; *H. influenzae *– n – 26

TMP-SMX exposure: More than 1 organism per subject

Organisms not included – Coagulase negative staphylococcus – 10; Streptococcus viridans – 2; other commensals – 11; no growth – 10; contaminants – 3

*# *p = 0.0104 (Fisher's Exact 2-tail test)

¶p = 0.048 (Fisher's Exact 2-tail test)

Risk factors for colonization by predominant organisms are shown in Table 3. For Enterobacteriaceae, age below a year (OR 4.4 [1.9–10.9]) and CDC Stage C disease (OR 3.6 [1.5–8.6]) were significant factors on multivariate analysis. For *S. aureus*, having moderate or severe CD4 depletion was associated with colonization (OR 3.4 (1.3–11.8]). There was a trend towards decreased carriage of gram-negative respiratory organisms in subjects on TMP-SMX at baseline (OR 0.3 [0.09–0.9] p = 0.057) and also with a relative lack of CD4 depletion (CDC Immunological Class 2 or 3: OR 0.3 [0.1–0.9] p = 0.005). No risk factors were identified for *S. pneumoniae *carriage.

**Table 3:** Risk factors for colonization by selected bacteria.

Organism	Odds ratio (95% CI)	Bivariate p-value	Multivariate logistic regression	Wald p-values
Enterobacteriaceae				
Age < 1 year	4.6 (2–11.1)	0.0004	4.4 (1.9–10.9)	0.0008
Stage C disease	3.7 (1.6–8.6)	0.002	3.6 (1.5–8.6)	0.005
Female	0.5 (0.23–1.19)	0.13		
CDC Immunological Class 2 or 3	2 (0.7–6.9)	0.23		
Weight for age-Z-score <-2	1.6 (0.7–3.5)	0.27		
No prior TMP-SMX at baseline	0.3 (0.03–1.3)	0.22		
Present and/or previous hospitalization	1.1 (0.4–3.7)	0.9		
*S. aureus*	1 (0.3–3.8)	1		
Age < 1 year	1.2(0.6–2.4)	0.6		
Stage C	0.1 (0.6–2.9)	0.4		
Weight for age-Z-score <-2	2 (1–3.9)	0.051	1.7 (0.8–3.5)	0.11
CDC Immunological Class 2 or 3	3.7 (1.4–12.9)	0.007	3.4 (1.3–11.8)	0.029
Female	1.2 (0.63–2.4)	0.54		
No prior TMP-SMX at baseline	0.97 (0.42–2.1)	0.94		
Present and/or previous hospitalization	2.04 (0.8–6.3)	0.14		
*S. pneumoniae*				
Age < 1 year	0.53 (0.2–1.1)	0.09		
Stage C	0.51 (0.2–1.6)	0.13		
Weight for age-Z-score <-2	0.71 (0.4–1.4)	0.31		
CDC Immunological Class 2 or 3	0.8 (0.4–1.6)	0.5		
Female	0.97 (0.5–1.9)	0.92		
No prior TMP-SMX at baseline	1.4 (0.4–5.3)	0.67		
Present and/or previous hospitalization	0.64 (0.29–1.46)	0.28		
Gram-negative respiratory organisms				
Age < 1 year	0.53 (0.2–0.98)	0.055	0.59 (0.24–1.33)	0.22
Stage C	0.8 (0.3–1.7)	0.51		
Weight for age-Z-score <-2	0.46 (0–0.9)	0.029	0.55 (0.25–1.2)	0.14
CDC Immunological Class 2 or 3	0.32 (0.16–0.66)	0.002	0.33 (0.15–0.71)	0.005
Female	1.4 (0.75–2.8)	0.28		
No prior TMP-SMX at baseline	5.1 (1.4–22.1)	0.058	0.33 (0.09–0.94)	0.057
Present and/or previous hospitalization	0.5 (0.23–1.12)	0.094	0.63 (0.27–1.54)	0.3

Multivariate logistic regression only where p ≤ 0.1 on univariate analyses

N = 203

Antibiotic resistance patterns are shown in table 4. One hundred and forty-two (88%) of 160 isolates tested, were resistant to TMP-SMX. A high percentage of organisms were resistant to first line antibiotics used for severe community acquired infections. Seventy-seven percent of *S. aureus *were MRSA and 81% were resistant to gentamicin. For *S. pneumoniae*, 20 of 48 (42.7%) were resistant to penicillin by oxacillin screening and one (2.1%) was resistant to cefotaxime. MIC's for penicillin were determined for 18 isolates, of which 7 (38.9%) were fully sensitive (MIC ≤ 0.06 μg/ml), 9 (50%) had intermediate resistance (MIC 0.12 – 1 μg/ml) and 2 (11.1%) had high level resistance (MIC ≥2 μg/ml). Sixteen of 32 (50%) Enterobacteriaceae produced extended-spectrum beta-lactamase (ESBL), thus resistant to third generation cephalosporins. Eighteen (56%) were resistant to gentamicin and 5 (15.6%) to amikacin.

**Table 4:** NP isolates and resistance to TMP-SMX and other selected antimicrobials

**Organisms isolated**	**Resistant to TMP-SMX**	**Resistance to selected antibiotics**
*S. pneumoniae*	43 (89.6%)	Pen 20 (41.7%)# CTX 1 (2.1%)
Gram negative respiratory		
*H. influenzae*	20 (80%)	Amp 3 (12%); Amox/clav 1 (4%)
*M. catarrhalis*	2 (18.2%) Δ	† Beta-lactamase +ve 12 (85.7%)
*S. aureus*	40 (91%)	Clox – 34 (77.3%) Gent 36 (81.8%), Amik – 0
Enterobacteriaceae	29 (90.6%)	CTX 16 (50%); Gent 18 (56%); Amik 5 (15.6%); PTZ 10 – 31.3%) Mero – 0
Pseudomonas	0	Gent – 1 (20%); Amik – 0

Clox – cloxacillin, Gent – gentamicin, Amik – amikacin, Pen – penicillin; CTX – cefotaxime; Amp – ampicillin, Amox/clav – amoxicillin-clavulanate

# *S. pneumoniae *– MIC performed in 18 isolates: 7 (38.9%) fully sensitive (MIC ≤ 0.06 μg/ml) 9 (50%) with intermediate resistance (MIC 0.12 – 1 μg/ml) and 2 (11.1%) with high level resistance (MIC ≥2 mg/ml) to penicillin

*M. catarrhalis*: Δ 11 isolates tested for MIC; † 14 of 17 isolates tested for beta-lactamase production. No significant differences for resistance to penicillin, cloxacillin, cefotaxime, gentamicin, amikacin and TMP-SMX by present or previous exposure to TMP-SMX

The influence of baseline status of TMP-SMX on resistance is shown in Table 5. Baseline levels of resistance to TMP-SMX were high, regardless of status but there was a trend for higher resistance to TMP-SMX in subjects receiving it at baseline (p = 0.065). MRSA was significantly associated with baseline TMP-SMX (87% of subjects; p = 0.002) even though baseline resistance in those never having received TMP-SMX was also high (70%).

**Table 5:** Relationship between TMP-SMX status at baseline and antibiotic resistance in NP isolates

**Antibiotic**	**Total isolates tested**	**Not on TMP-SMX at baseline **Resistance (%)	**On TMP-SMX at baseline **Resistance (%)	**Previously received TMP-SMX **Resistance (%)	**P-value**
TMP-SMX	151	31 (86.1%)	93 (91.2%)	9 (69.2%)	0.065
Cloxacillin	44	7 (70%)	27 (87.1)	0	0.002
Cefotaxime	32	7 (53.9%)	9 (50%)	0	0.58

In subjects without a history of previous or current hospitalization, antibiotic resistance in the most common organisms isolated was not reduced. Unexpectedly, for *S. pneumoniae*, TMP-SMX resistance was significantly more common in subjects *without *a history of hospitalization. (Table 6)

**Table 6:** Current or previous hospitalization: relationship to selected NP organisms and antibiotic resistance

Organism	No current or previous hospitalization (% organisms)	Antibiotic resistance (% tested for selected antibiotic)	P-value
*S. aureus*	5 (11.4%)	Cloxacillin – 4 (80%)	1
		TMP-SMX – 5 (100%)	1
*S. pneumoniae*	12 (25%)	Penicillin – 7 (58.3%)	0.2
		TMP-SMX – 8 (66.7%)	0.011
Enterobacteriaceae	6 (18.8%)	Cefotaxime 3 (50%)	1
		Gentamicin 6 (50%)	0.5
		TMP-SMX 6 (83.3%)	0.48

Fisher's Exact 2-tail test

Bacteremias are shown in Table 7. Nineteen subjects (9.4%) had 23 episodes of bacteremia. Four had two episodes and three had two pathogens from a single blood culture. The bacteremias occurred a median of 30.3 (IQ: 4.6 – 36.6) weeks post enrolment. Only one subject had bacteremia prior to enrolment (3 days). Enterobacteriacae were the most commonly isolated group. More than 50% produced ESBL and were also resistant to gentamicin, showing a similar profile to NP isolates. Reduced susceptibility to penicillin occurred in 3 of 4 *S. pneumoniae *isolates.

**Table 7:** Blood culture isolates and antibiotic resistance patterns

**Organisms isolated**	**Number**	**Resistant to TMP-SMX**	**Resistance to selected antibiotics**
*S. pneumoniae*	4	1 (only 1 tested)	Pen sensitive 1; intermediate resistance 2; resistant 1
Gram negative respiratory *H. influenzae*	2	2 (100%)	Amp 0 (0%)
S. aureus*	3	2 (66.6%)	Clox – 2 (77.3%) Gent 2 (81.8%), Amik – 0
Enterobacteriacae†	13	8 (72.3%)	CTX – 6 (54.5%); Gent – 6 (54.5%); Amik – 1 (10%); PTZ -10 (31.3%) Mero – 0
Acinetobacter	1	0	CTX – 0; gent – 0
Pseudomonas	1	1	PTZ -1; Gent – 1; Amik – 1
*Shigella flexneri*	1	1	Amp – 0; gent -1

*No data for one isolate

† Two isolates had no data for for cefotaxime, gentamicin and TMP-SMX; Three had no data for amikacin

Clox – cloxacillin; Gent – gentamicin; Amik – amikacin; Pen – penicillin; Amp – ampicillin; CTX – cefotaxime; PTZ – piperacillin/tazobactam; Mero – meropenem

Three of four subjects with the same organism isolated from nasopharynx and blood had identical resistance patterns. One had ESBL-producing and gentamicin-resistant *K. pneumoniae*. Another had Enterobacter, sensitive to 3^rd ^generation cephalosporins and gentamicin and a third had *S. pneumoniae *with intermediate resistance to penicillin. The fourth subject had MRSA in the nasopharynx but no sensitivity information for the blood culture isolate.

## Discussion

Our study confirms a high carriage of potential pathogens, many of which are antibiotic resistant, from NP and blood culture isolates in HIV-infected children. The majority of organisms from both NP and blood were resistant to TMP-SMX and other antibiotics commonly used for community-acquired infection. A disturbing finding was the high prevalence of MRSA and ESBL-producing Enterobacteriaceae from both sites.

The demographic features of HIV-infected children are similar to those described previously at the Tygerberg Family clinic where the majority were under two years of age, had moderate or severe immunosuppression and symptomatic HIV disease [23]. Mortality has been described elsewhere and was not analyzed further as it may have been affected by multiple factors beyond the scope of this report [17]. The significant difference in age of subjects stratified by TMP-SMX exposure probably reflects utilization of the public health service. Subjects entered the study from January 2003, as the Vertical Transmission Prevention program was gradually being introduced in the Western Cape. Consequently, many of the older infants and children were only identified through knowledge of their parents' HIV status, or when presenting with clinical disease suggestive of HIV. Those never exposed to TMP-SMX were significantly younger than those already on TMP-SMX. Those previously on the drug but not receiving it at baseline were older, possibly reflecting poor retention in the public health system.

Colonization by *S. pneumoniae*, *H. influenzae *and *M. catarrhalis *is well documented in early childhood [6]. Approximately 50% of children carry *S. pneumoniae *at two years of age, declining to 20% by 7 years. Below 7 years of age 40% of immunocompetent children in developed countries carry *M. catarrhalis *and 30% *H. influenzae *[24]. Socioeconomic factors such as housing, overcrowding, poor hygiene, access to health care and daycare are major determinants of colonization. For example, in Australia, Aboriginal children are colonized with *M. catarrhalis*, *H. influenzae *and *S. pneumoniae *by 20 days of age, whereas non-Aboriginal children acquire these organisms after 200 days of age [25]. Increased numbers of colonizing faecal organisms such as Enterobacteriaceae and Pseudomonas have been found in malnourished infants and children from resource-poor settings in the pre-HIV era [7,8]. A recent comparative community-based study of children aged between 4 months and 5 years, showed more frequent carriage of Gram-negative enteric pathogens in Brazilian (50%) and Angolan (57%) than Dutch children (4%) [26].

Studies on NP *S. pneumoniae *in HIV-infected adults and children from developed countries showed similar carriage rates to uninfected subjects [27,3,28]. In Kenya, Rusen *et al *found no increased NP *S. pneumoniae *colonization in asymptomatic HIV-infected infants (20%) compared to seronegative controls (22%) [5].

In our study, colonization by Enterobacteriaceae was associated with age below a year and advanced HIV disease. *S. aureus *was linked to CD4 depletion and Gram-negative respiratory organisms to absence of CD4 depletion. High carriage rates of *S. aureus *and possible relationship with invasive disease, has been noted in HIV-infected adults [29,30]. In a study from the Western Cape, comparing community acquired pneumonia in HIV-infected and uninfected infants, Zar *et al *found a higher rate of *S. aureus *in nasopharyngeal aspirates and invasive disease, linking this finding to TMP-SMX prophylaxis in HIV-positive children [15]. Similarly, in a recent survey of children hospitalized with severe pneumonia in Kwazulu-Natal, South Africa, HIV-infected children were significantly more likely to be colonized with *S. aureus *(31%) than HIV-uninfected children (13.8%) [2].

TMP-SMX resistance occurs commonly in children. For example in a recent survey of *H. influenzae *colonization in daycare attendees in Brazil, 46% were resistant to TMP-SMX and 10% had multidrug resistance [31]. Although not reported, HIV prevalence was likely to be extremely low. Abdel-Haq showed that both young age and TMP-SMX prophylaxis were risk factors for NP colonization with multiply resistant *S. pneumoniae *in immunocompromised and immunocompetent children from Detroit, USA [14].

Antibiotic resistance was extremely common in our study. Between 80 – 90% of most pathogens (except for *M catarrhalis*) were resistant to TMP-SMX. Almost 80% of *S. aureus *were methicillin-resistant and 50% of Enterobacteriaceae had ESBL production. The majority of children had advanced disease and frequent contact with both in- and outpatient hospital facilities. High prevalence of antibiotic resistance has already been described in malnourished children with pneumonia and also in children with hospital-associated bacterial infection [32,33]. We did not record TMP-SMX usage or frequency of healthcare utilization in other family members, but speculate that both were likely to be high, as other family members are also likely to be HIV-infected. The children enrolled in the study come from poor socio-economic conditions where overcrowding and spread of antibiotic resistant pathogens in homes are likely. As we did not link previous hospitalization with antibiotic resistance in NP organisms, we speculate that antibiotic resistance is well established in homes of study subjects and possibly in the communities, as well. As evidence of this, TMP-SMX resistance was significantly higher in *S. pneumoniae *in subjects without a history of hospitalization. Also, even though we found significantly more MRSA in children on TMP-SMX at baseline, 70% of those not on TMP-SMX also had MRSA, supporting the contention that the organism is well established in the homes of the infants. A major limitation of our study is that we did not document NP colonization in HIV-uninfected children from the same communities or within the household of our subjects. We plan to address these issues.

Colonization with antibiotic resistant organisms has been noted in surveys of African children and adults. In 1997, Woolfson described *S. pneumoniae *in 72% of 260 Zambian school children < 6 years of age. Antibacterial resistance occurred in 34.1% of isolates; intermediate resistance to penicillin occurred in 14.3% and to TMP-SMX in 12.7% [34]. In the Kenyan study from 1997, Rusen *et al *documented that 60% of *S. pneumoniae *isolates had intermediate resistance to penicillin [5]. Both studies preceded the widespread use of TMP-SMX for chemoprophylaxis in HIV-infected infants [35]. MRSA has been found more commonly in HIV-infected than uninfected children in Kwazulu-Natal, South Africa [2].

The incidence of bacteremia (9.4%) is higher that from a survey in the pre-HIV era. In a study of hospitalized children at Tygerberg Hospital in 1989, 2% had bacteremia, the most frequent isolates being *S. pneumoniae *and *S. aureus*. In community-acquired infections, pathogens were also sensitive to first line antibiotics [36]. In a study of infants with severe lower respiratory tract infection in Soweto, *S. pneumoniae *and *S. aureus *from bacteremic infants showed significantly higher levels of penicillin (54 versus 23%) and methicillin resistance (60% versus 0%) respectively in HIV-infected than uninfected infants [13].

Community-associated infection due to MRSA is an emerging problem in children from developed countries [37,38]. In contrast, community-associated infection due to ESBL-producing organisms is well documented in adults, but not yet in children [39].

The colonizing NP flora and high level of antibiotic resistance for Enterobacteriaceae and *S. aureus *suggest that empiric antibiotic treatment should be adapted to cover MRSA and ESBL-producing Enterobacteriaceae. Also, amikacin is a more appropriate aminoglycoside than gentamicin for severely ill HIV-infected children in our region. A limitation of our study, however, is that we did not distinguish between community and hospital acquired bacteremia or nasopharyngeal colonization. To our knowledge, this is the first documentation of ESBL-producing Enterobacteriaceae causing bacteremia or colonizing the nasopharynx in HIV-infected children.

## Conclusion

The majority of HIV-infected children were colonized with pathogenic organisms, with high levels of antibiotic resistance. These included respiratory isolates such as *S. pneumoniae*, *H. influenzae *and *M. catarrhalis*, and gram-negative organisms such as Enterobacteriaceae and Pseudomonas. The spectrum of antibiotic resistance in blood culture isolates is similar to that found in the nasopharynx. These findings have implications for empiric antibiotic therapy in sick HIV-infected children. We recommend antibiotics appropriate for ESBL-producing Enterobacteriaceae and MRSA in severely ill HIV-infected children in our setting. However, there is also concern that widespread use of broader spectrum agents may lead to resistance to these agents as well. There is an urgent need to further document the spectrum and antibiotic resistance profiles of HIV-infected children in under-resourced communities.

## Competing interests

The authors declare that they have no competing interests.

## Authors' contributions

MC was the co-principal investigator of the original study and contributed to the design of the present study. He wrote the manuscript and conducted the analyses. EW supervised the microbiology, assisted with original analyses and gave intellectual input to the manuscript. JS performed all the microbiological assays, and wrote the initial draft of the manuscript. AW assisted with bacteraemia data and gave intellectual input to the manuscript. HZ co-designed the original study and the present study. She gave intellectual input to the manuscript and analyses. JS was awarded a M. Med degree at Stellenbosch University for work in this study.

All co-authors read and approved the final version of the manuscript.

## Pre-publication history

The pre-publication history for this paper can be accessed here:


